# Structural and functional evaluation of the peripheral vasculature in patients with PAD using MRI

**DOI:** 10.1186/1532-429X-17-S1-P406

**Published:** 2015-02-03

**Authors:** Erin K Englund, Michael C Langham, Emile R Mohler, Thomas F Floyd, Felix W Wehrli

**Affiliations:** 1 University of Pennsylvania, Philadelphia, PA, USA; 2 Stony Brook University, Stony Brook, NY, USA

## Background

Impaired vascular reactivity in patients with peripheral artery disease (PAD) may be due to primary microvascular disease, limited macrovascular supply, or a mixture of these two factors. The combination of structural and functional MR-based evaluation of the peripheral vasculature may provide insight into the mechanism of impairment. The objective of this study is to investigate preliminary results of a combined protocol to assess both microvascular function via perfusion imaging, and macrovascular patency and the presence of collateralization via a non-contrast angiography technique in patients with PAD.

## Methods

In this IRB-approved study with ongoing recruitment, 19 patients with PAD (mean ± SD of the ankle-brachial index (ABI) = 0.62 ± 0.15) were scanned using a functional and structural MR protocol at 3T. The leg with the lower ABI was scanned. For the functional evaluation, perfusion in the calf was continuously measured throughout an ischemia-reperfusion paradigm using a variant of a pulsed arterial spin labeling (PASL) MRI sequence. PASL data were post-processed to yield dynamic time courses of perfusion in regions of interest in the gastrocnemius, soleus, peroneus, and tibialis anterior muscles. From the time courses, the time to peak perfusion (TTP) and peak hyperemic flow (PHF) were determined for each muscle. To evaluate macrovascular structure of the popliteal trifurcation and distal, Quiescent-Interval Single-Shot (QISS), a 2D multi-slice cardiac-gated non-contrast MR angiography (MRA) technique, was used to acquire images in the same leg (calf only).

## Results

**Figure 1:**
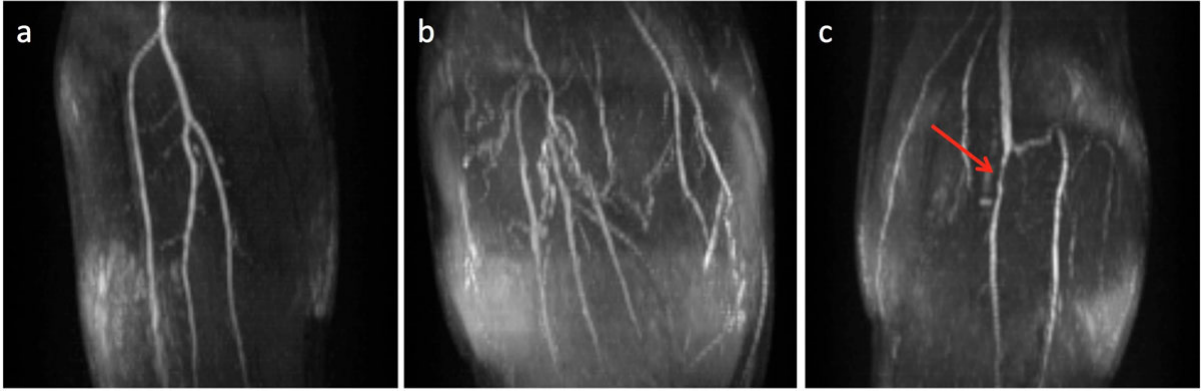
Example MRAs of the leg: (a) patient with patent popliteal trifurcation (ABI = 0.63, TTP = 94 s, PHF = 15 mL/min/100g), (b) patient with extensive collateralization throughout the calf (ABI = 0.81, TTP = 48 s, PHF = 37 mL/min/100g), and (c) patient with stenosis (arrow) in anterior tibial artery (ABI = 0.67, TTP = 60 s, PHF = 38 mL/min/100g).

**Figure 2:**
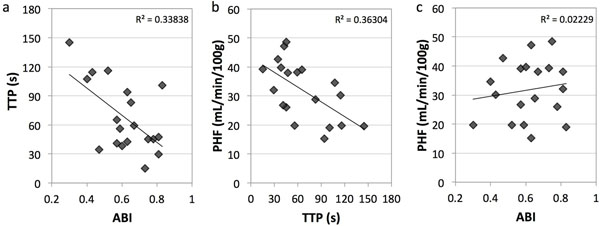
Significant correlations were found between TTP and ABI, and between TTP and PHF, however no relationship was found between ABI and PHF.

Example MRAs of characteristic findings are shown in Figure 1. Several patients showed marked collateralization, however the perfusion TTP for any muscle was not noticeably affected. In one subject, a stenosis in the anterior tibial artery, the artery that supplies both the tibialis anterior and peroneus muscles, was detected, however TTP was approximately equal to TTP in the gastroc and soleus muscles. Average TTP is inversely correlated with ABI (p <0.01) and with average PHF (p<0.01), however no correlation was detected between ABI and PHF (Figure 2).

## Conclusions

In the patients examined, preliminary results suggest that a reduction of reactive hyperemia response time may be independent of the presence macrovascular lesions in the popliteal trifurcation. These results do not preclude upstream macrovascular involvement, though they could also suggest that functional reactivity of the vasculature is primarily mediated by the microvasculature. However, it is most likely that the microvascular and macrovascular involvements both contribute to the measured impairment in vascular reactivity. Future scans will include acquisition of a full peripheral MRA in addition to the high-resolution MRA of the calf, allowing us to further investigate the relationship between structural impairment and functional response of the peripheral vasculature.

## Funding

This work was supported by an award from the American Heart Association and NIH Grants R01 HL075649, R01 HL109545.

